# microRNA-485 inhibits the malignant behaviors of retinoblastoma by directly targeting Wnt3a

**DOI:** 10.3892/or.2022.8347

**Published:** 2022-06-14

**Authors:** Xueman Lyu, Ling Wang, Jia Lu, Hong Zhang, Lina Wang

Oncol Rep 41: 3137–3147, 2019; DOI: 10.3892/or.2019.7061

Following the publication of the above paper, the authors submitted a request to the Editorial Office to retract the article on account of the fact that the ethical approval for the tumor xenograft experiments performed in mice, as shown in Fig. 7, had not been obtained from their hospital; consequently, all authors were in agreement that the article should be retracted to ensure the integrity of the scholarly record. Independently of this request, it was also drawn to the Editor's attention by a concerned reader that data in several of the figures were strikingly similar to data appearing in different form in other articles by different authors.

Owing to the fact that there were contentious data in the above article that had already been published prior to its submission to *Oncology Reports*, and after assessing the authors' request, the Editor concurs that this paper should be retracted from the Journal. The Editor apologizes to the readership for any inconvenience caused.

